# Evaluating the biodistribution for [^68^Ga]Ga-PSMA-11 and [^18^F]F-PSMA-1007 PET/CT with an inter- and intrapatient based analysis

**DOI:** 10.1186/s13550-024-01097-3

**Published:** 2024-04-05

**Authors:** Cristina E. Popescu, Boya Zhang, Thomas Sartoretti, Noel Spielhofer, Stephan Skawran, Jakob Heimer, Michael Messerli, Alexander Sauter, Martin W. Huellner, Philipp A. Kaufmann, Irene A. Burger, Alexander Maurer

**Affiliations:** 1 Department of Nuclear Medicine, Cantonal Hospital Baden, Baden, Switzerland; 2 Department of Health Sciences and technology, ETH Zurich, Zurich, Switzerland; 3 Department of Nuclear Medicine, University Hospital Zurich, University of Zurich, Zurich, CH-8091 Switzerland; 4 Department of Mathematics, Seminar for Statistics, ETH Zurich, Zurich, Switzerland; 5 Department of Radiology, University Hospital Tuebingen, Tuebingen, Germany

**Keywords:** Radioligand therapy, PSMA-PET/CT, Therapy selection, Liver uptake, F18-PSMA-1007, Ga68-PSMA-11

## Abstract

**Background:**

Liver uptake in [^68^Ga]Ga-PSMA-11 PET is used as an internal reference in addition to clinical parameters to select patients for [^177^Lu]Lu-PSMA-617 radioligand therapy (RLT). Due to increased demand, [^68^Ga]Ga-PSMA-11 was replaced by [^18^F]F-PSMA-1007, a more lipophilic tracer with different biodistribution and splenic uptake was suggested as a new internal reference. We compared the intra-patient tracer distribution between [^68^Ga]Ga-PSMA-11 and [^18^F]F-PSMA-1007.

**Methods:**

Fifty patients who underwent PET examinations in two centers with both [^18^F]F-PSMA-1007 and [^68^Ga]Ga-PSMA-11 within one year were included. Mean standardized uptake values (SUV_mean_) were obtained for liver, spleen, salivary glands, blood pool, and bone. Primary tumor, local recurrence, lymph node, bone or visceral metastasis were also assessed for intra- and inter-individual comparison.

**Results:**

Liver SUV_mean_ was significantly higher with [^18^F]F-PSMA-1007 (11.7 ± 3.9) compared to [^68^Ga]Ga-PSMA-11 (5.4 ± 1.7, *p* < .05) as well as splenic SUV_mean_ (11.2 ± 3.5 vs.8.1 ± 3.5, *p* < .05). The blood pool was comparable between the two scans. Malignant lesions did not show higher SUV_mean_ on [^18^F]F-PSMA-1007. Intra-individual comparison of liver uptake between the two scans showed a linear association for liver uptake with SUV_mean_ [^68^Ga]Ga-PSMA-11 = 0.33 x SUV_mean_ [^18^F]F-PSMA-1007 + 1.52 (*r* = .78, *p* < .001).

**Conclusion:**

Comparing biodistribution of [^68^Ga]Ga and [^18^F]F tracers, liver uptake on [^68^Ga]Ga-PSMA-11 PET is the most robust internal reference value. Liver uptake of [^18^F]F-PSMA-1007 was significantly higher, but so was the splenic uptake. The strong intra-individual association of hepatic accumulation between the two scans may allow using of a conversion factor for [^18^F]F-PSMA-1007 as a basis for RLT selection.

**Supplementary Information:**

The online version contains supplementary material available at 10.1186/s13550-024-01097-3.

## Introduction

Despite excellent diagnostic tools for prostate cancer imaging and availability of several effective therapy options, metastatic castration-resistant prostate cancer (mCRPC) is associated with a poor prognosis [1, 2]. Internal radioligand therapy (RLT) is an exciting development in the field of prostate cancer treatment and has become increasingly important over the past five years. With phase II and III trials of RLT targeting prostate-specific membrane antigen (PSMA) with [^177^Lu]Lu-PSMA-617 in mCRPC patients showing positive results, RLT is now an approved therapeutic option for those who progress after chemotherapy [3, 4]. For this VISION trial, patients were selected according to the therapeutic concept using [^68^Ga]Ga-PSMA-11 PET/CT in addition to clinical parameters [3]. Eligible patients had to be free of suspicious lesions with a [^68^Ga]Ga-PSMA uptake equal to or less than that of the liver parenchyma and have at least one lesion that absorbed more tracer than the liver. The Australian TheraP trial also used a combination of [^68^Ga]Ga-PSMA-11 and [^18^F]-FDG PET/CT for patient selection [4]. Therefore, all randomized phase II/III studies to date have evaluated PSMA distribution based on [^68^Ga]Ga-PSMA-11.

[^68^Ga] is a generator product with a maximum of two to three doses per elution, reducing the potential quantity of examinations. The relatively short half-life of 68 minutes further limits the geographic distribution. The increased demand for PSMA imaging quickly led to a massive shortage. An alternative was sought and found in [^18^F]F-based tracers such as [^18^F]F-PSMA-1007. Their longer half-life (109 min), the higher production capacity in the cyclotron (up to 20 doses per production) and the better properties of [^18^F]F compared to [^68^Ga]Ga led to a switch to [^18^F]F-PSMA-1007 in many institutions in Switzerland. Another advantage of [^18^F]F-PSMA-1007 for the detection of local recurrences is a slightly different biodistribution compared to [^68^Ga]Ga-PSMA-11: the tracer is excreted hepatically rather than renally [5]. This feature may have better diagnostic efficacy for detecting local recurrence in the bladder area [6, 7]. On the other hand, it reduces the detection of liver metastases due to the significantly higher liver background and has an increased incidence of indeterminate findings (e.g. unspecific bone uptake) [8–10]. Nevertheless, the use of [^18^F]F-PSMA-1007 for patient selection in [^177^Lu]Lu-PSMA-617 RLT remains a topic of ongoing debate in clinical practice.

The purpose of this retrospective bicentric study was to determine whether the uptake of [^18^F]F-PSMA-1007 in the spleen approximates the activity of [^68^Ga]Ga-PSMA-11 in the liver of the same patient and can thus be used as an internal surrogate reference for therapy selection. In addition, we wanted to evaluate whether the salivary glands could alternatively serve as a more robust reference organ.

## Methods

### Study design and population

**Fig. 1:**
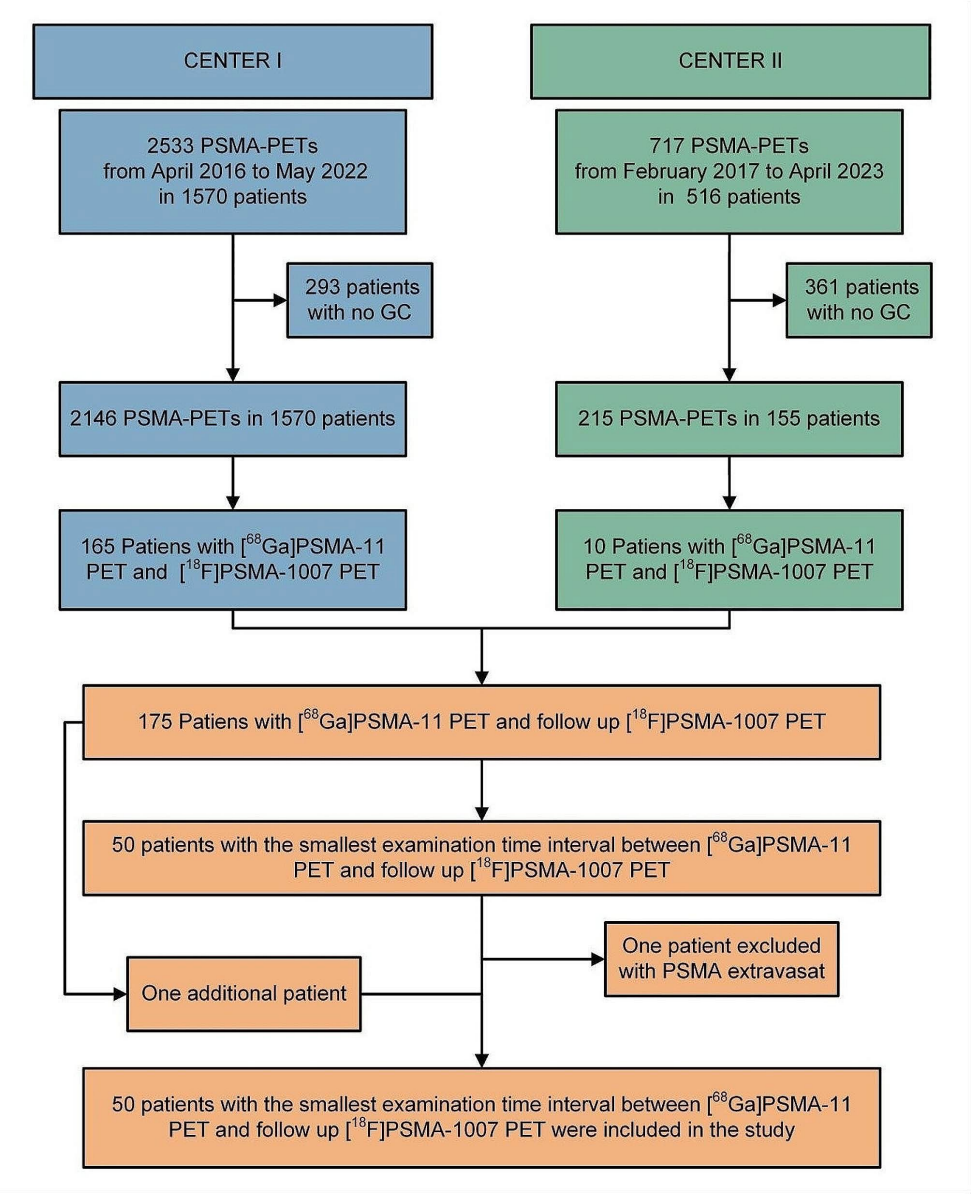
Study flow chart. GC, general consent

In this retrospective bicentric study, we included 50 patients from two centers (Center I and Center II) who underwent [^68^Ga]Ga-PSMA-11 PET/CT and [^18^F]F-PSMA-1007 PET/CT scans between December 2018 and April 2023. Patients who underwent both scans were included regardless of indication and tumor stage. From the entire cohort, 50 patients with the shortest time interval between [^68^Ga]Ga-PSMA-11 PET/CT and [^18^F]F-PSMA-1007 PET/CT were selected as study population. In cases of poor image quality or tracer extravasation, patients were excluded from the study and replaced to achieve the final sample size of 50 patients. The study flow chart is shown in Fig. 1.

The following patient information was collected: age, weight, injected tracer dose, and PSA levels not older than four weeks at the time of PSMA-PET. The indications for the PSMA PET scans were also recorded (staging, biochemical recurrence, response assessment).

The study was conducted in accordance with ICH-GCP guidelines and the Declaration of Helsinki. Informed consent was obtained from all patients and the protocol was approved by the local ethics committee (KEK 2023 − 00812).

#### Imaging protocols

PSMA-PET/CT was performed using a Discovery MI scanner (GE Healthcare, Waukesha, WI), Discovery 690 Standard scanner (GE Healthcare), Discovery VCT scanner (GE Healthcare), Discovery ST scanner (GE Healthcare), or Siemens Biograph mCT Flow (Siemens Healthineers, Munich, Germany). The injected dose was 3–4 MBq/kg for [^18^F]F-PSMA-1007 and 2–3 MBq/kg for [^68^Ga]Ga-PSMA-11 at both centers. The maximum injected dose was not more than 350 MBq. The uptake time was 60 min for [^68^Ga]Ga-PSMA-11 and 90 min for [^18^F]F-PSMA-1007 at both centers.

#### Image analysis

**Fig. 2:**
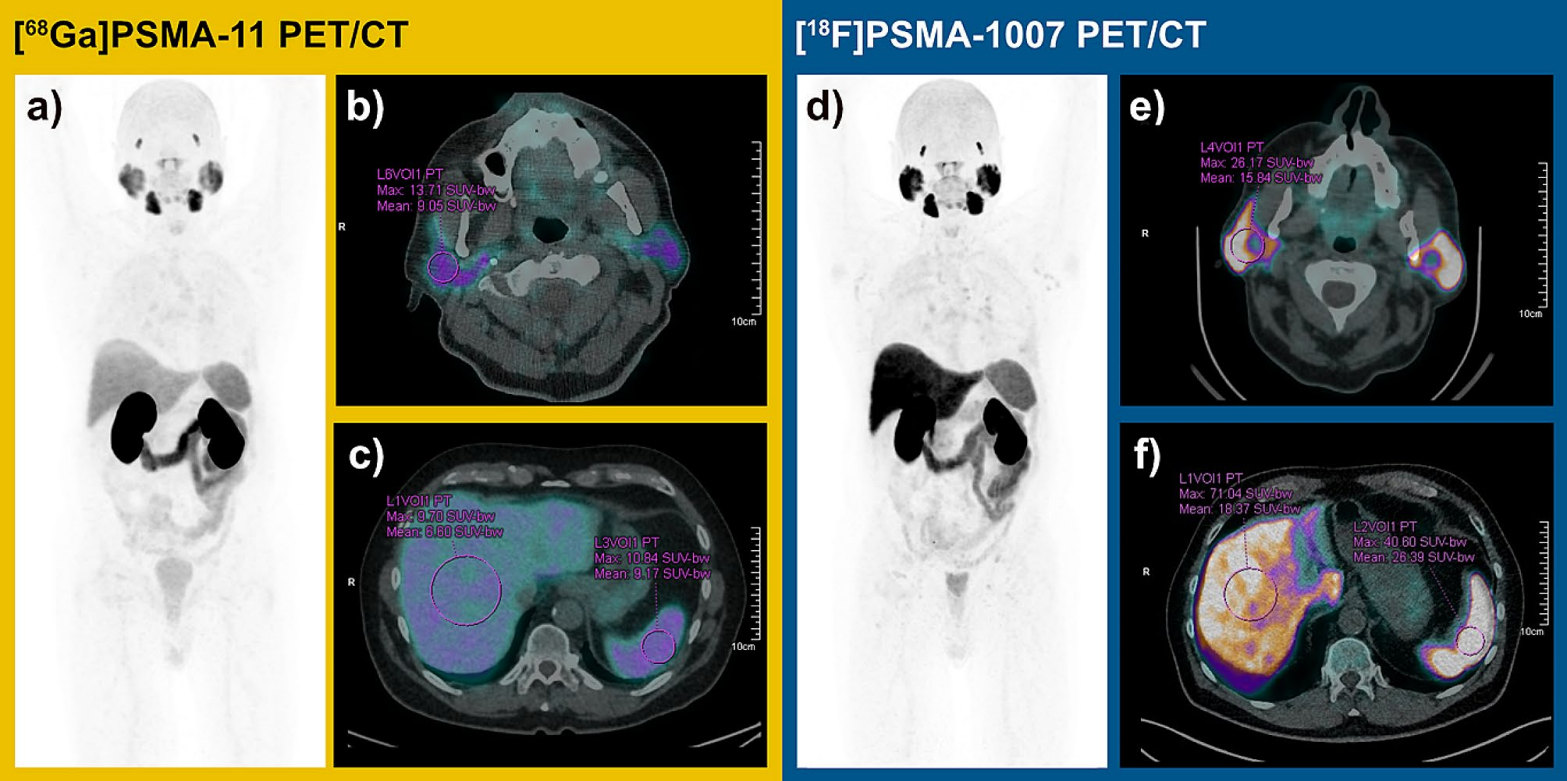
Illustration of one patient with both [^68^Ga]Ga-PSMA-11 PET/CT and [^18^F]F-PSMA-1007 PET/CT examinations within twelve months for rising PSA (PSA 10 ng/ml in [^68^Ga]Ga-PSMA-11 PET/CT and PSA 13 ng/ml in [^18^F]F-PSMA-1007 PET/CT). (**a**) Whole-body maximum intensity projection (MIP) image in [^68^Ga]Ga-PSMA-11 PET with (**b**) SUV measurement in the salivary gland and (**c**) in the liver parenchyma in axial fused [^68^Ga]Ga-PSMA-11 PET/CT. (**d**) MIP image in [^18^F]F-PSMA-1007 PET. (**e**) SUV measurement in the parotid gland and (**f**) in liver parenchyma in axial fused [^18^F]F-PSMA-1007 PET/CT

Image analysis was performed centrally by a nuclear medicine physician using standardized volumes of interest (VOIs) for different organs placed within physiological uptake. Standardized uptake values (SUV) such as SUV_max_ and SUV_mean_ were obtained for liver, spleen, salivary glands, blood pool and bone. Blood pool values were measured in the ascending aorta and bone values were measured in the fifth lumbar vertebra. An example of the measurement is shown in Fig. 2. If present, additional VOIs were placed over the primary tumor, the side of local recurrence, or the most active lymph node, bone, or visceral metastasis to measure SUV_max_. To exclude a significant reduction in normal uptake due to large tumor volumes as described by Gafita et al., we measured PSMA volume using an absolute threshold at SUV 4.0 in all patients with multiple or large lesions [11].

### Statistical analysis

Continuous variables were summarised as median and interquartile range (IQR) or mean and standard deviation (SD), whereas categorical variables were presented as counts and percentages. Normality of variables was assessed using the D’Agostino-Pearson test. Depending on the distribution of the variables, paired / unpaired samples t-tests or rank sum tests were used to compare the data sets. Waterfall plots and Bland-Altman plots were used for distribution analysis of SUV_mean_ in different organs. For Bland-Altman plots, limits of agreement (BA-LA) and bias were calculated. Statistical analyses were performed for different anatomical regions: liver, spleen, salivary glands, blood and bone. The aim was to explore potential systematic differences in SUV_mean_ values between two tracers within different organs. Linear mixed models were used with SUV_mean_ as the response variable and the tracers as predictors. To account for the inherent variability within patients, a random intercept for each patient ID was added to the models. This adjustment effectively fit the models with a paired t-test, while allowing the intraclass correlation coefficient (ICC) to be examined. This coefficient, represents the ratio of random intercept variance to total residual variance and elucidates the proportion of residual variance attributable to patient-specific factors. Graphical analysis of the residual distributions revealed variance homogeneity. To assess unequal variances between tracers, Levene tests were performed on the SUV_mean_ values for each organ system. To derive a correction value, a simple linear regression was performed on the [^68^Ga]Ga-PSMA-11 values (response) and [^18^F]F-PSMA-1007 SUV_mean_ values (predictor) specifically for the liver SUV_mean_ data, with a Tukey-Anscombe plot for residual analysis. For all hypothesis tests an alpha of 0.05 was used.

Statistical analysis was performed with R (version 4.3.2, R Foundation for Statistical Computing) and MedCalc Statistical Software version 19.1 (MedCalc Software bv, Ostend, Belgium).

## Results

### Patient characteristics

Patient characteristics are presented in Table 1. No significant difference between weight (*p* = .888), and PSA value (*p* = .321) was found in the [^18^F]F-PSMA-1007 and [^68^Ga]Ga-PSMA-11 cohorts. As most patients received the [^18^F]F-PSMA-1007 after the [^68^Ga]Ga-PSMA-11 scan, the age was significantly higher in the [^18^F]F-PSMA-1007 cohort. The median PSA level was 3.1 ng/mL in both cohorts. The mean injected dose was 239 MBq (IQR 208–265 MBq) for [^18^F]F-PSMA-1007 PET and 141 MBq (IQR 121–154 MBq) for [^68^Ga]Ga-PSMA-11 PET. The clinical indications for [^68^Ga]Ga-PSMA-11 PET and [^18^F]F-PSMA-1007 PET are shown in Table 2. The mean time interval between the corresponding [^68^Ga]Ga-PSMA-11 PET and [^18^F]F-PSMA-1007 PET was 8.9 ± 3.2 months. In seven patients a [^68^Ga]Ga-PSMA-11 was performed for staging, followed with a [^18^F]F-PSMA-1007 for PSA persistence after a mean time of 7.4 ± 2.7 months. Forty-three patients received both PSMA PET scans for biochemical recurrence with a mean time of 9.2 ± 3.2 months between. In one patient, the second PSMA PET scan was performed to assess post-treatment response at an interval of 12.0 months.

**Table 1:** Patient characteristics

Patient characteristics	[^68^Ga]Ga-PSMA-11 median (IQR)	[^18^F]F-PSMA-1007 median (IQR)	p-value
age [y]	74.0 (67.0–77.0)	75.0 (68.0–77.9)	< 0.05
weight [kg]	85.0 (75.0–98.0)	82.5 (72.0–98.0)	0.888
dose [MBq]	140.5 (121.0–153.6)	239.0 (208.0–265.0)	< 0.05
PSA [ng/ml]	3.1 (0.9–11.0)	3.1 (0.6–11.6)	0.312

Prostate specific Antigen (PSA).

**Table 2:** Indications for PSMA-PET

PSMA-PET indications	[^68^Ga]Ga-PSMA-11 n (%)	[^18^F]F-PSMA-1007 n (%)
staging	7 (14)	0
biochemical recurrence	42 (84)	49 (98)
post-treatment response	1 (2)	1 (2)

### Distribution of physiological PSMA uptake in different organs

**Fig. 3:**
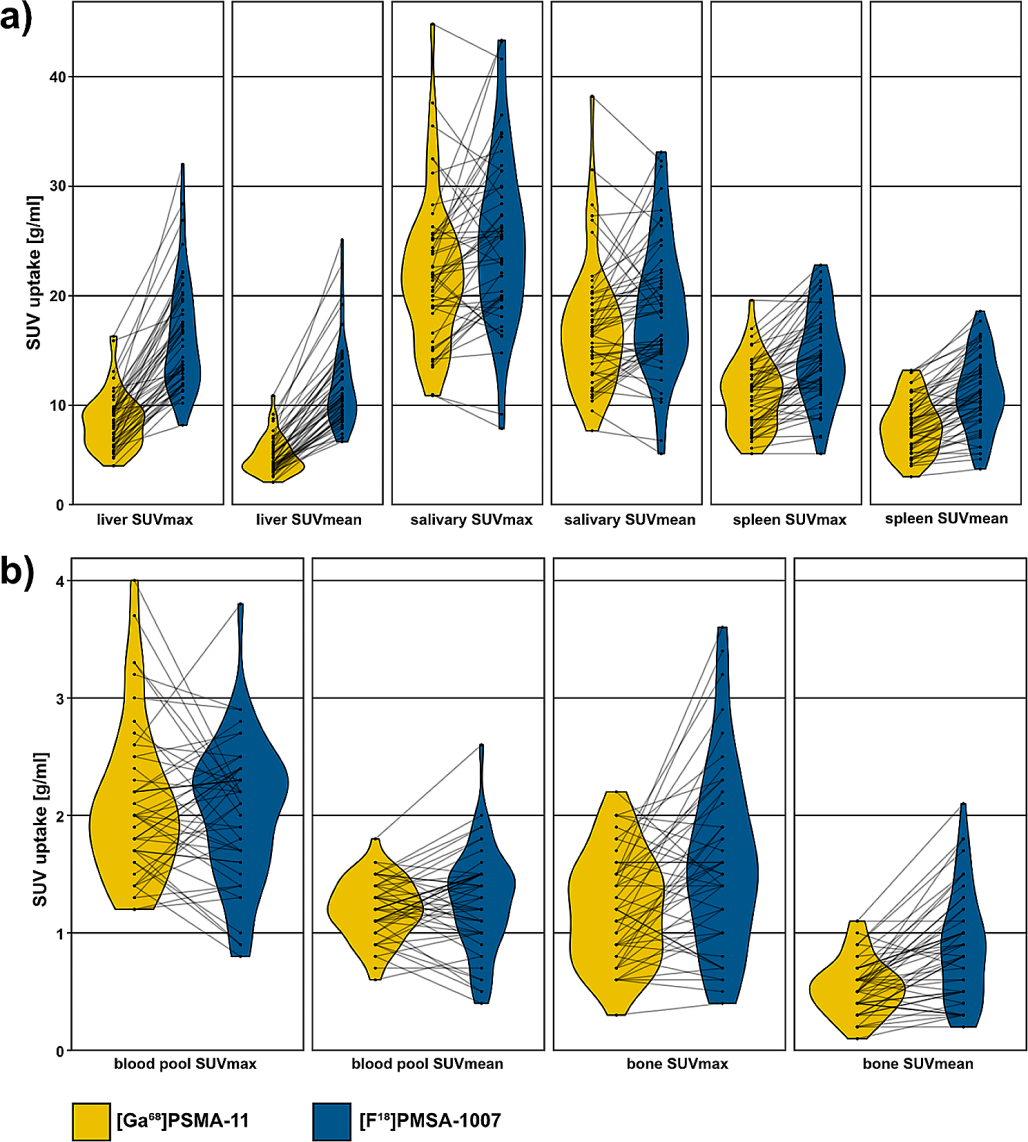
Violin plots of physiological tracer uptake (SUV_mean_ and SUV_max_) in the corresponding [^68^Ga]Ga-PSMA-11 PET and [^18^F]F-PSMA-1007 PET for (**a**) liver, spleen and salivary glands and (**b**) for blood pool and bone

**Fig. 4:**
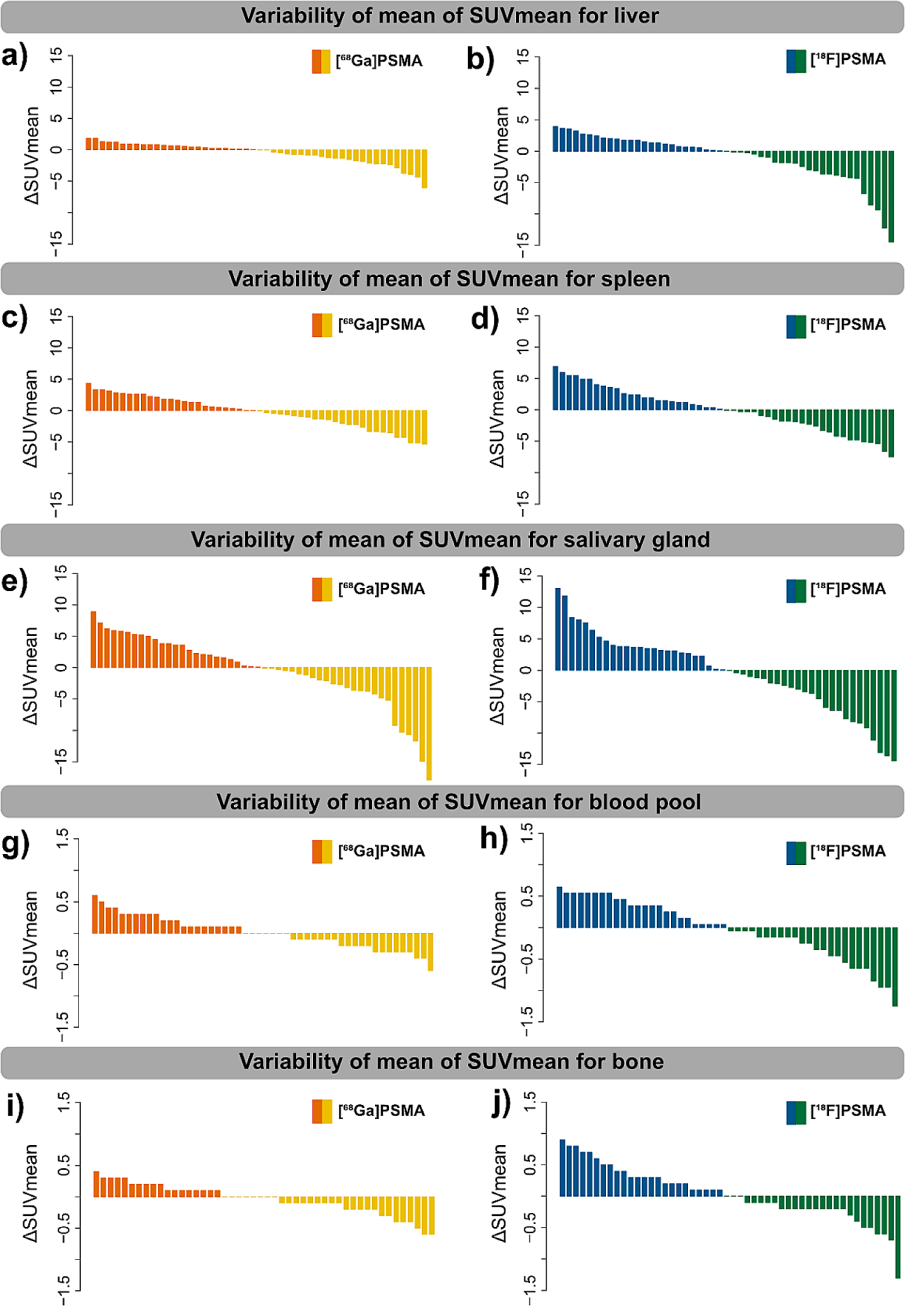
Illustration of the heterogeneity of tracer uptake between patients for normal organs with waterfall plots, showing the difference between the SUV_mean_ value for each patient and the averaged SUV_mean_ of the cohort, for [^68^Ga]Ga-PSMA-11 PET and [^18^F]F-PSMA-1007 PET, respectively (**a**) and (**b**) for the liver, (**c**) and (**d**) for the spleen, (**e**) and (**f**) for the salivary gland, (**g**) and (**h**) for the blood pool, (**i**) and (**j**) for the bone

SUV_mean_ for liver, spleen, salivary gland and bone were significantly higher with [^18^F]F-PSMA-1007 PET compared to [^68^Ga]Ga-PSMA-11 PET (*p* < .05). Only the SUV_mean_ for the blood pool showed no significant difference (*p* < .153). The results are shown in Table 3. The splenic uptake of [^18^F]F-PSMA-1007 in the same patient, is significantly higher than the hepatic uptake of [^68^Ga]Ga-PSMA-11 (*p* < .0001). In Fig. 3, the measured SUV values (SUV_max_ and SUV_mean_) are graphically shown in violin plots with lines connecting measures from one patient. The waterfall plots in Fig. 4 show the interpatient variability for all organs for both [^68^Ga]Ga-PSMA-11 PET and [^18^F]F-PSMA-1007 PET. Correlation analysis using Bland-Altman plots with Limits of Agreement (BA-LA) [^68^Ga]Ga-PSMA-11 PET as the reference standard and the variation of [^18^F]F-PSMA-1007 revealed for liver (BA-LA -0.7 to -11. 8, bias of -6.3), spleen (BA-LA 0.7 to -6.8), salivary gland (BA-LA 7.5 to 11.3, bias of -1.9), bone (0.6 to -0.8, bias of -0.07) and blood pool (0.41 to -1.09, bias of -0.34) (Supplement 1). Random intercept/interindividual heterogeneity analysis shows that SUV_means_ for spleen and salivary gland are much more patient-dependent (Supplement 2). The intraclass correlation coefficient was 56% for liver, 81% for spleen, 69% for salivary gland, 50% for blood pool and 47% for bone. Levene tests revealed significantly higher variances for the SUV_mean_ in the [^18^F]F-PSMA-1007 group compared to the [^68^Ga]Ga-PSMA-11 PET group for liver (15.37, 2.79, *p* < .01), spleen (12.28, 6.33, *p* < .05), blood (0.18, 0.06, *p* < .01), and bone (0.22, 0.06, *p* < .001).

**Table 3:** Physiological PSMA uptake (SUV_mean_) for liver, spleen, salvary glands, blood pool or bone

Organ	SUV_mean_ [^68^Ga]Ga-PSMA-11 mean ± SD	SUV_mean_ [^18^F]F-PSMA-1007 mean ± SD	p-value
liver	5.4 ± 1.7	11.7 ± 3.9	< 0.05
spleen	8.1 ± 2.5	11.2 ± 3.5	< 0.05
salivary glands	17.2 ± 5.9	19.11 ± 6.2	< 0.05
blood pool	1.2 ± 0.3	1.3 ± 0.4	0.153
bone	0.5 ± 0.2	0.9 ± 0.5	< 0.05

Only three patients had extensive disease with a volume of more than 530 ml, two of them extensive on both scans, one patient only on [^18^F]F-PSMA-1007 PET. Thus a systemic underestimation of normal uptake due to a tumor sink effect can be excluded in this cohort.

### Regression analysis between [^68^Ga]Ga-PSMA-11 PET and [^18^F]F-PSMA-1007 PET for liver uptake

**Fig. 5:**
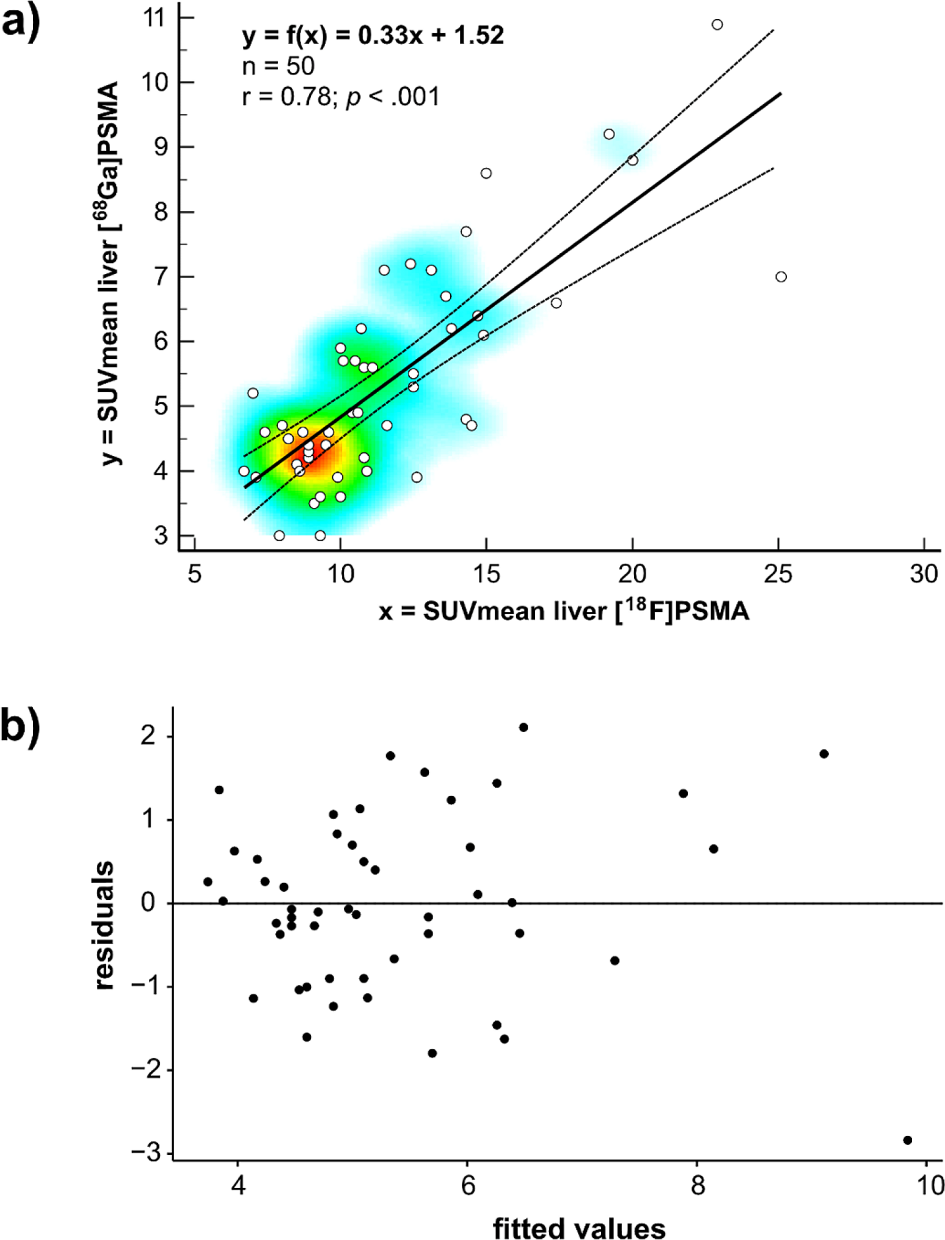
Correlation and regression between SUV_mean_ liver uptake on corresponding [^68^Ga]Ga-PSMA-11 PET and [^18^F]F-PSMA-1007 PET. (**a**) Linear regression model allows to calculated the correlation between SUV_mean_ liver values for [^68^Ga]Ga-PSMA-11 PET and [^18^F]F-PSMA-1007 PET with the following formula: SUV_mean_[^68^Ga]Ga-PSMA-11 = 0.33 x SUV_mean_[^18^F]F-PSMA-1007 + 1.52 (*r* = .78, *p* < .001). (**b**) Tukey-Anscom plot for residual analysis showing no systematic bias and narrow limits of agreement

There was a high correlation between [^68^Ga]Ga-PSMA-11 PET and [^18^F]F-PSMA-1007 on an intra-patient basis (*p* < .001). A linear regression model allows the calculation of a conversion factor for SUV_mean_ liver values between [^68^Ga]Ga-PSMA-11 PET and [^18^F]F-PSMA-1007 PET: SUV_mean_ [^68^Ga]Ga-PSMA-11 = 0.33 x SUV_mean_ [^18^F]F-PSMA-1007 + 1.52 (*r* = .78, *p* < .001) (Fig. 5a). The Graphical analysis of residuals showed an acceptable residual distribution (Fig. 5b).

### [^68^Ga]Ga-PSMA-11 and [^18^F]F-PSMA-1007 uptake in malignant lesions

**Fig. 6:**
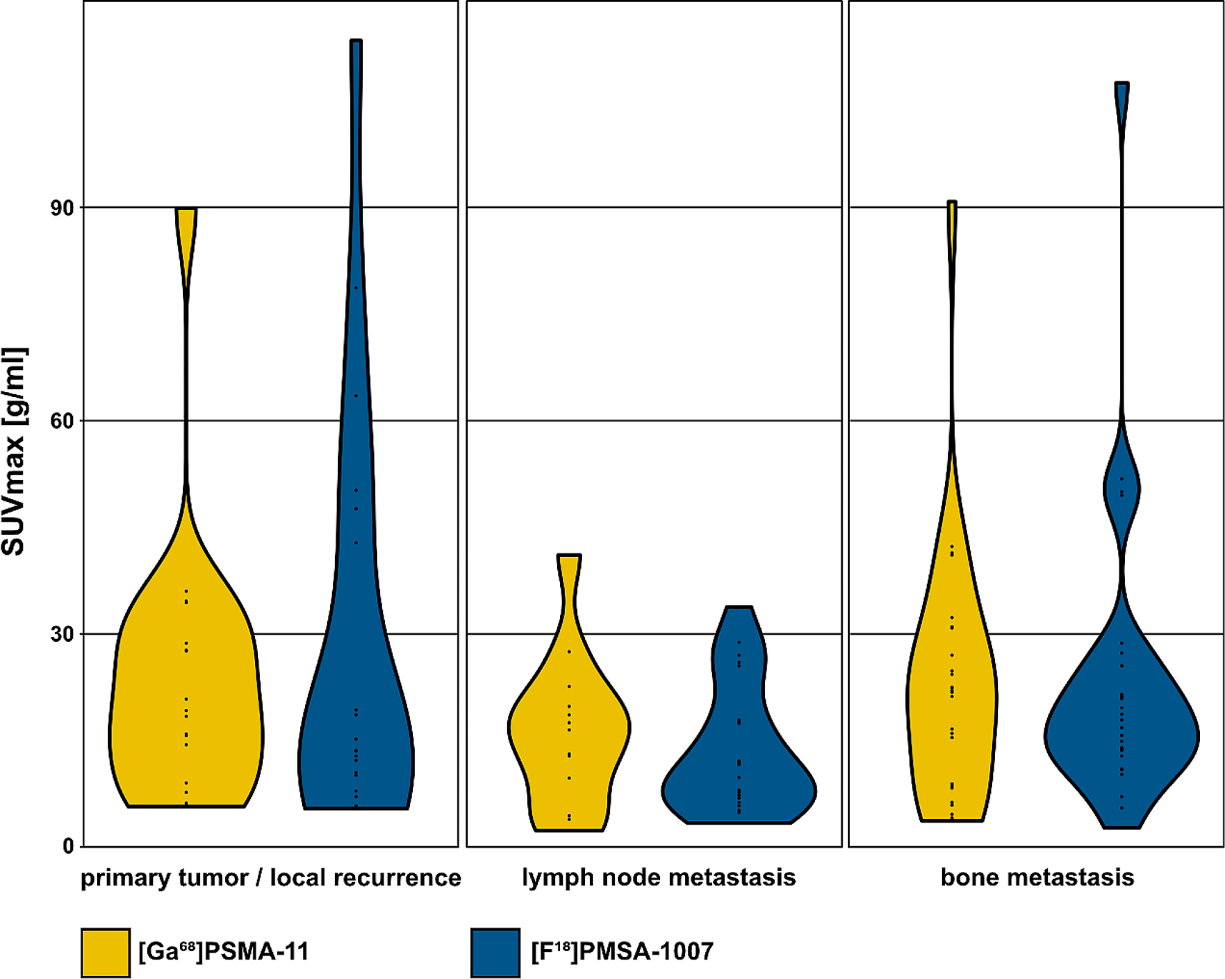
Violin plots of PSMA uptake (SUV_max_) in malignant lesions in [^68^Ga]Ga-PSMA-11 PET and [^18^F]F-PSMA-1007 PET for primary tumors / local recurrences, lymph node metastasis and bone metastasis. This is not an intra-lesion comparison with the same lesions on both scans, but just giving an overview of uptake distribution for malignant lesions for both scans. No difference between uptake for [^68^Ga]Ga-PSMA-11 PET and [^18^F]F-PSMA-1007 PET was found (*p* < .05)

On [^68^Ga]Ga-PSMA-11 PET, 19 (38%) patients had PSMA-positive primary/local recurrence, 26 (52%) had PSMA-positive lymph node metastases, and 13 (26%) had PSMA-positive bone metastases. With [^18^F]F-PSMA-1007, 19 (38%) patients had PSMA-positive primary / local recurrence, 29 (58%) had PSMA-positive lymph node metastases and 19 (38%) had PSMA-positive bone metastases. There was no difference between the SUV_max_ of the hottest malignant lesions (primary/local recurrence, lymph node metastases, bone metastases) between the two tracers. These results are shown in Table 4. The distribution of the SUV_max_ is shown in violin plots (Fig. 6). Visceral metastases were present in three patients (6%) on the [^68^Ga]Ga-PSMA-11 PET and in four patients (8%) on the [^18^F]F-PSMA-1007 PET scans.

**Table 4:** Pathological PSMA uptake (SUV_max_) for primary tumor / local recurence, and the most active metastasis

Malignant lesions	SUV_max_ [^68^Ga]Ga-PSMA-11 mean ± SD	SUV_max_ [^18^F]F-PSMA-1007 mean ± SD	p-value
primary tumor / local recurrence	23.5 ± 19.0	28.9 ± 29.7	0.343
lymph node metastasis	32.1 ± 20.3	22.9 ± 18.2	0.484
bone metastasis	16.2 ± 10.6	13.6 ± 8.9	0.646

## Discussion

The present study was a retrospective comparison of the intra-patient tracer distribution between [^68^Ga]Ga-PSMA-11 and [^18^F]F-PSMA-1007. The study confirmed the expected significant higher hepatic uptake for [^18^F]F-PSMA-1007 compared to [^68^Ga]Ga-PSMA-11 due to its higher lipophilicity, leading to hepatic excretion. We also showed that within the same patient, the splenic uptake of [^18^F]F-PSMA-1007 is significantly higher than the hepatic uptake of [^68^Ga]Ga-PSMA-11.

Replacing the liver by the spleen as internal reference for [^177^Lu]Lu-PSMA-617 RLT enrollment could therefore lead to a shift in patient selection. Furthermore, the inter-individual range of splenic uptake is higher for both [^18^F]F-PSMA-1007 and [^68^Ga]Ga-PSMA-11 compared to hepatic PSMA-uptake. This may be one of the reasons why Seifert et al. excluded 42% of patients in a cohort with either [^18^F]F-PSMA-1007 or [^68^Ga]Ga-PSMA-11 scans based on VISION trial selection criteria for patients scanned with [^18^F]F-PSMA-1007 using the spleen as internal reference [12]. The higher SUV_mean_ of the internal reference organ spleen in [^18^F]F-PSMA-1007 may shift patients away from [^177^Lu]Lu-PSMA-617 RLT compared to the drop-out rate of only 13% in the VISION study based on hepatic uptake in [^68^Ga]Ga-PSMA-11. Whether this leads to a similar patient selection as the cut-off used in [^68^Ga]Ga-PSMA-11 studies has never been tested systematically. The internal reference value for liver uptake with [^18^F]F-PSMA-1007 would exclude a large number of patients from RLT who would likely be included with [^68^Ga]Ga-PSMA-11. Other authors have compared physiological uptake in 14 individuals with PSA recurrence using both [^68^Ga]Ga-PSMA-11 and [^18^F]F-DCFPyL PET/CT, showing a marginal increase in liver parenchymal uptake with [^18^F]F-DCFPyL (SUV_mean_ 6.2 vs. 5.1, *p* = .005) [6].

**Fig. 7:**
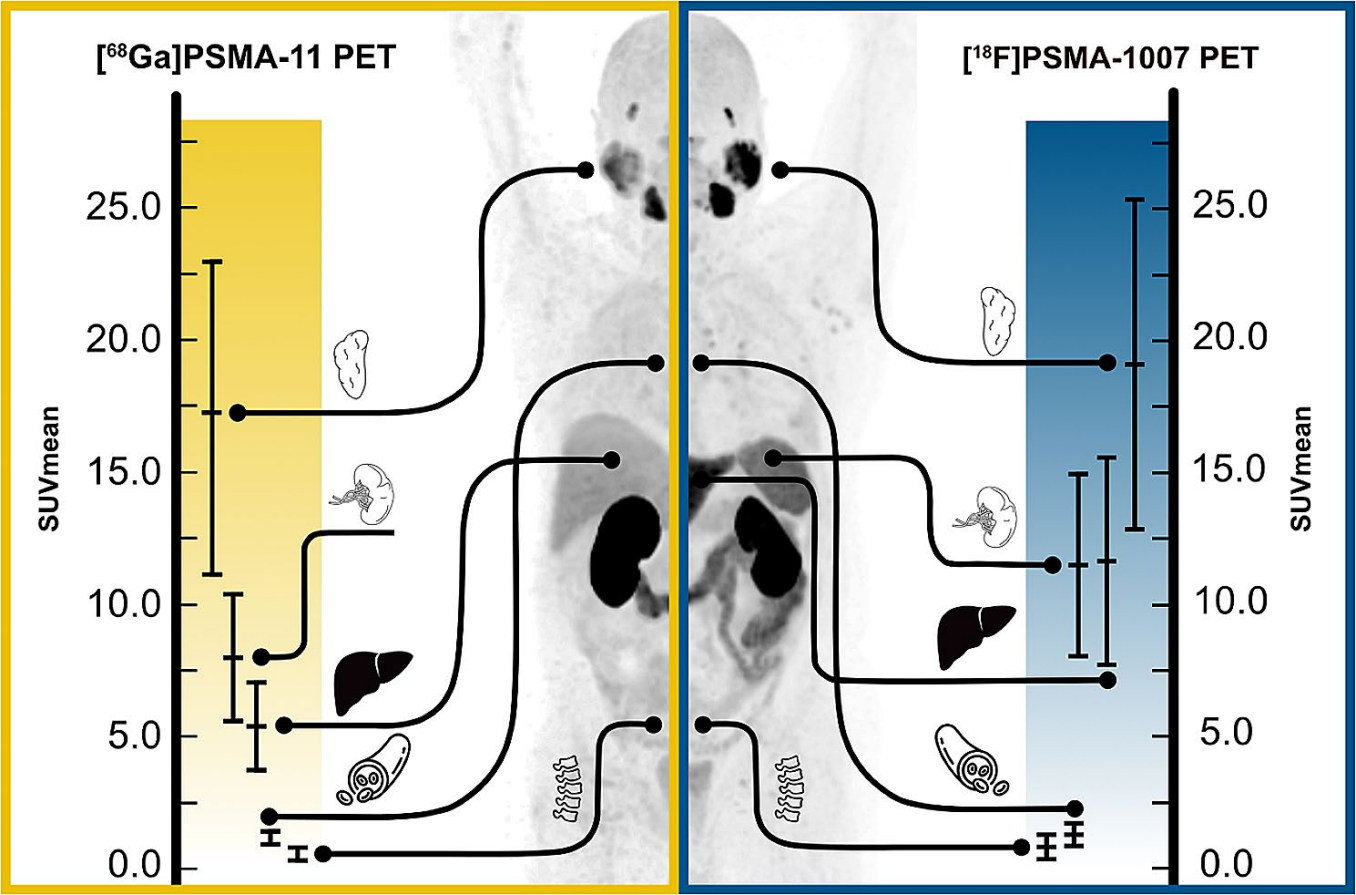
Graphical representation of SUV_mean_ with mean and standard deviation for the parotid gland, spleen, liver, blood pool and bone for both [^68^Ga] Ga-PSMA-11 PET and [^18^F] F-PSMA-1007 PET.

Some studies have suggested using the salivary glands as an internal reference, but also based on [^68^Ga]Ga-PSMA-11 images, reporting that patients in whom more than 80% of the lesions have uptake higher than the salivary glands benefit from treatment [13]. Notably, salivary gland uptake showed better overall comparability between the two tracers; however, it exhibited the highest intra- and inter-individual variability of all examined organs. This may limit the use of the proposed salivary gland score as a robust internal reference [13]. However, the use of [^18^F]F-PSMA-1007 in clinical practice for patient selection in [^177^Lu]Lu-PSMA-617 RLT is still under discussion. A comprehensive graphical representation of the main study results is shown in Fig. 7.

To determine the potential influence of increased tumor volume on conventional tracer distribution in the present study, we quantified tumor volume. Only three patients were classified as having high-volume disease, defined as greater than 530 ml according to the criteria of Gafita et al. [11].

Although direct intra-lesion comparisons were not performed, the SUV_max_ of the most PSMA-active malignant PCA lesions showed comparability between the two scans. In particular, there was no evidence of generalized increased [^18^F]F-PSMA-1007 accumulation in primary tumors, local recurrences, lymph nodes or bone metastases. This observation differs from the prospective intra-individual analysis performed by Pattison et al., who reported a significantly higher uptake of [^18^F]F-PSMA-1007 in lymph nodes (mean SUV_max_ 11.1 vs. 8.7) [14]. However, our results are consistent with their observation that PSMA uptake in bone metastases was similar (mean SUV_max_ 30.9 vs. 30.7) [14].

Both [^68^Ga]Ga-PSMA-11 and [^18^F]F-PSMA-1007 PET tracers are commonly used, depending on considerations such as local accessibility, practicality, imaging quality, and the specific clinical context. In particular, [^18^F]F-PSMA-1007 may have diagnostic efficacy in assessing primary tumors [9, 14, 15] or identifying local recurrence [7, 10, 16] due to reduced urinary excretion. Conversely, the use of [^18^F]F-PSMA-1007 has been associated with an increased incidence of indeterminate findings, particularly in the bone, termed non-specific bone uptake, and celiac ganglia [8, 10].

This study has several limitations. First, due to the retrospective nature of the study the scans were performed at two different time points and in some cases for different indications (staging, biochemical recurrence, post-treatment response), this is precluding a direct intra-lesion analysis and therefore makes a comparison of uptake in malignant very limited. However, careful efforts were made to mitigate the temporal differences between the two scans, with no discernible differences in PSA levels at the time of imaging or in clinical indications. Second, a major limitation is the retrospective design, particularly given the heterogeneous nature of the patient cohort. Third, the current study cohort does not allow for a comprehensive assessment of patient eligibility for [^177^Lu]Lu-PSMA-617 RLT, as the majority of patients underwent imaging for staging or biochemical recurrence, limiting the scope of the study in this regard.

## Conclusion

Comparing biodistribution of [^68^Ga]Ga and [^18^F]F tracers, the most robust internal reference value is the liver uptake on [^68^Ga]Ga-PSMA-11 PET. As expected, the SUV liver on [^18^F]F-PSMA-1007 PET were significantly higher in comparison to [^68^Ga]Ga-PSMA-11 PET, but so was splenic uptake. The observed high intra-individual linearity in hepatic accumulation between the two tracers suggests the potential utility of a conversion formula for [^18^F]F-PSMA-1007 PET to provide a more equitable basis for RLT selection instead of the spleen as internal organ reference.

### Electronic supplementary material

Below is the link to the electronic supplementary material.

Supplementary Material 1

Supplementary Material 2

## Data Availability

The analyzed data may be available from the corresponding author upon reasonable request and with the permission of University Hospital Zurich, University of Zurich, Switzerland.

## References

[1] Rebello RJ, Oing C, Knudsen KE, Loeb S, Johnson DC, Reiter RE (2021). Prostate cancer. Nat Rev Dis Primers.

[2] Patell K, Kurian M, Garcia JA, Mendiratta P, Barata PC, Jia AY (2023). Lutetium-177 PSMA for the treatment of metastatic castrate resistant prostate cancer: a systematic review. Expert Rev Anticancer Ther.

[3] Sartor O, de Bono J, Chi KN, Fizazi K, Herrmann K, Rahbar K (2021). Lutetium-177-PSMA-617 for metastatic castration-resistant prostate Cancer. N Engl J Med.

[4] Hofman MS, Emmett L, Sandhu S, Iravani A, Joshua AM, Goh JC (2021). [(177)Lu]Lu-PSMA-617 versus cabazitaxel in patients with metastatic castration-resistant prostate cancer (TheraP): a randomised, open-label, phase 2 trial. Lancet.

[5] Giesel FL, Hadaschik B, Cardinale J, Radtke J, Vinsensia M, Lehnert W (2017). F-18 labelled PSMA-1007: biodistribution, radiation dosimetry and histopathological validation of tumor lesions in prostate cancer patients. Eur J Nucl Med Mol Imaging.

[6] Dietlein M, Kobe C, Kuhnert G, Stockter S, Fischer T, Schomacker K (2015). Comparison of [(18)F]DCFPyL and [ (68)Ga]Ga-PSMA-HBED-CC for PSMA-PET imaging in patients with relapsed prostate Cancer. Mol Imaging Biol.

[7] Dietlein F, Kobe C, Hohberg M, Zlatopolskiy BD, Krapf P, Endepols H (2020). Intraindividual comparison of (18)F-PSMA-1007 with Renally Excreted PSMA ligands for PSMA PET Imaging in patients with relapsed prostate Cancer. J Nucl Med.

[8] Grunig H, Maurer A, Thali Y, Kovacs Z, Strobel K, Burger IA, Muller J (2021). Focal unspecific bone uptake on [(18)F]-PSMA-1007 PET: a multicenter retrospective evaluation of the distribution, frequency, and quantitative parameters of a potential pitfall in prostate cancer imaging. Eur J Nucl Med Mol Imaging.

[9] Kroenke M, Mirzoyan L, Horn T, Peeken JC, Wurzer A, Wester HJ (2021). Matched-pair comparison of (68)Ga-PSMA-11 and (18)F-rhPSMA-7 PET/CT in patients with primary and biochemical recurrence of prostate Cancer: frequency of non-tumor-related uptake and Tumor Positivity. J Nucl Med.

[10] Rauscher I, Kronke M, Konig M, Gafita A, Maurer T, Horn T (2020). Matched-pair comparison of (68)Ga-PSMA-11 PET/CT and (18)F-PSMA-1007 PET/CT: frequency of pitfalls and detection efficacy in biochemical recurrence after radical prostatectomy. J Nucl Med.

[11] Gafita A, Wang H, Robertson A, Armstrong WR, Zaum R, Weber M (2022). Tumor Sink Effect in (68)Ga-PSMA-11 PET: myth or reality?. J Nucl Med.

[12] Seifert R, Telli T, Hadaschik B, Fendler WP, Kuo PH, Herrmann K (2023). Is (18)F-FDG PET needed to assess (177)Lu-PSMA Therapy Eligibility? A VISION-like, Single-Center Analysis. J Nucl Med.

[13] Hotta M, Gafita A, Murthy V, Benz MR, Sonni I, Burger IA (2023). PSMA PET tumor-to-salivary gland ratio to Predict Response to [(177)Lu]PSMA Radioligand Therapy: An International Multicenter Retrospective Study. J Nucl Med.

[14] Pattison DA, Debowski M, Gulhane B, Arnfield EG, Pelecanos AM, Garcia PL (2022). Prospective intra-individual blinded comparison of [(18)F]PSMA-1007 and [(68) Ga]Ga-PSMA-11 PET/CT imaging in patients with confirmed prostate cancer. Eur J Nucl Med Mol Imaging.

[15] Hoberuck S, Lock S, Borkowetz A, Sommer U, Winzer R, Zophel K (2021). Intraindividual comparison of [(68) Ga]-Ga-PSMA-11 and [(18)F]-F-PSMA-1007 in prostate cancer patients: a retrospective single-center analysis. EJNMMI Res.

[16] Dietlein F, Kobe C, Neubauer S, Schmidt M, Stockter S, Fischer T (2017). PSA-Stratified performance of (18)F- and (68)Ga-PSMA PET in patients with biochemical recurrence of prostate Cancer. J Nucl Med.

