# Supplementary figures and images for: Evaluating the biodistribution for [68Ga]Ga-PSMA-11 and [18F]F-PSMA-1007 PET/CT with an inter- and intrapatient based analysis
Source: EJNMMI Res. 2024 Apr 5;14:36. doi: 10.1186/s13550-024-01097-3 (PMC10997563; doi:10.1186/s13550-024-01097-3)

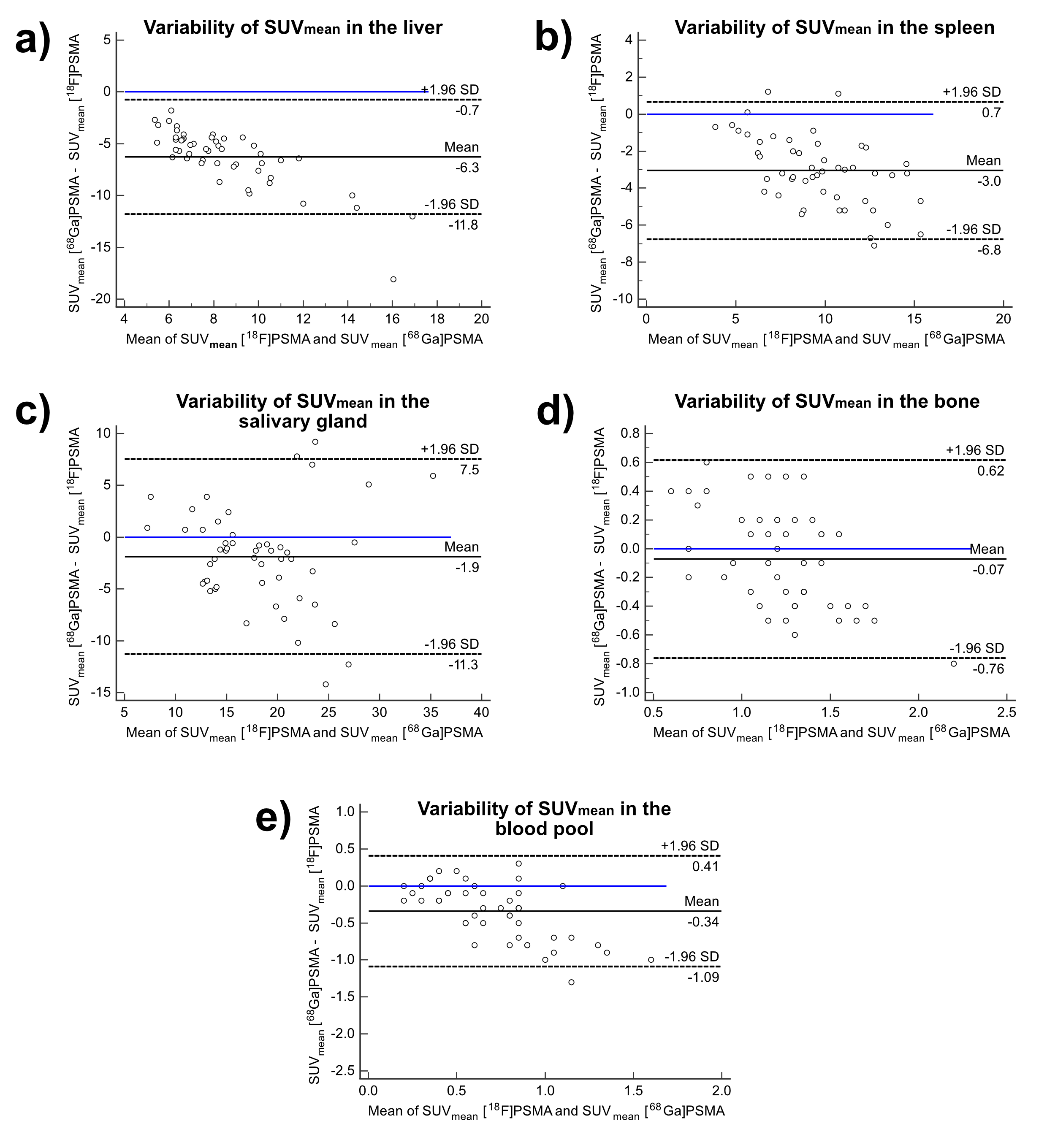

Supplement: Supplementary file 1 — Supplementary Material 1 [file 13550_2024_1097_MOESM1_ESM.tiff]

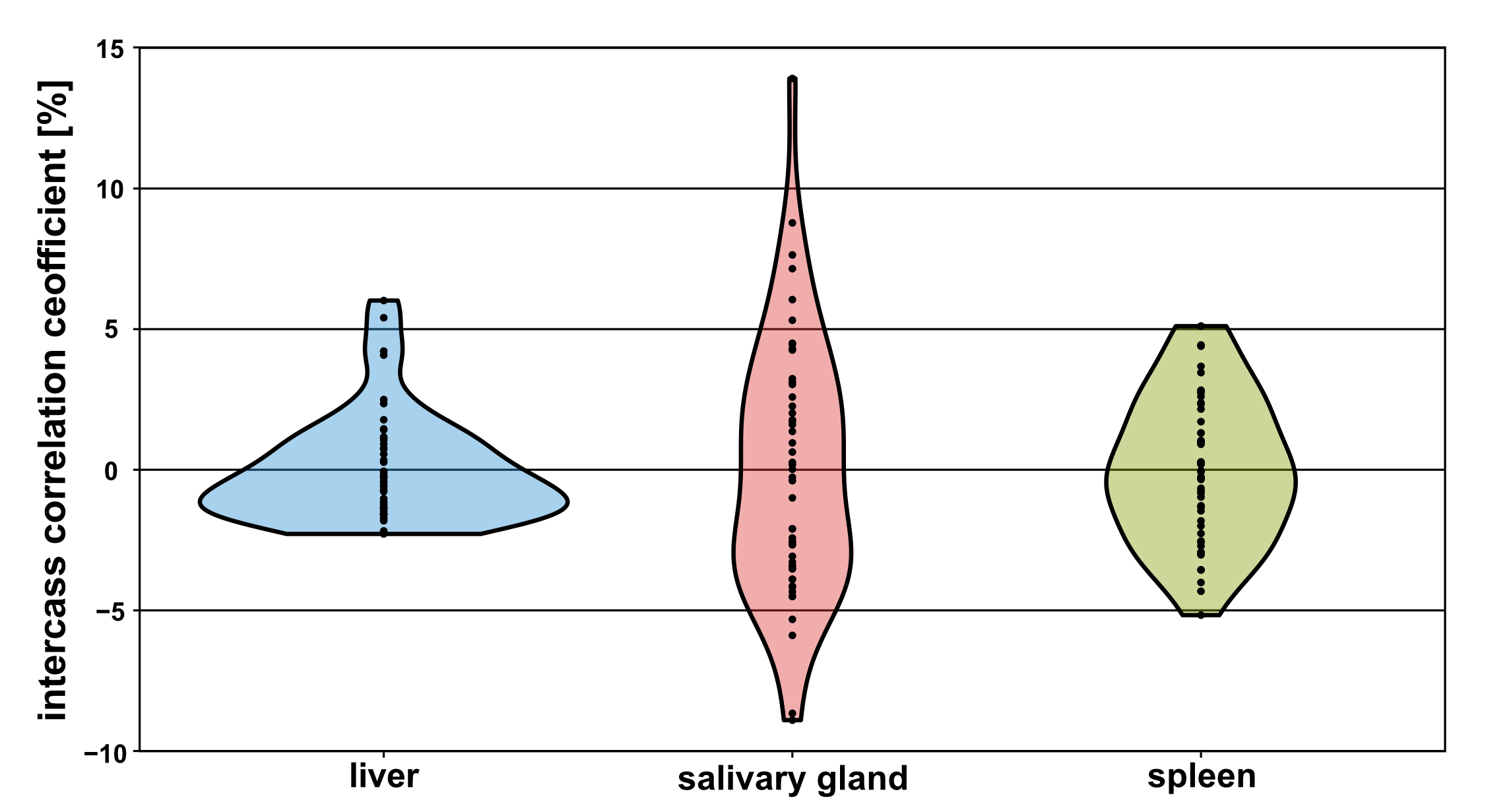

Supplement: Supplementary file 2 — Supplementary Material 2 [file 13550_2024_1097_MOESM2_ESM.tiff]
